# Effect of Mobile Phone Call Reminders With Standard Therapy Versus Standard Therapy Alone on Compliance With Iron Supplementation in Antenatal Women With Iron Deficiency Anemia: A Randomized Controlled Trial

**DOI:** 10.7759/cureus.29501

**Published:** 2022-09-23

**Authors:** Priya Sontakke, Kanchan S Dwidmuthe, Anjali Kawathalkar, Anuja Bhalerao

**Affiliations:** 1 Obstetrics and Gynaecology, N.K.P. Salve Institute of Medical Sciences and Research Centre, Nagpur, IND

**Keywords:** mobile phone call reminder, compliance, oral iron supplementation, pregnant women, iron-deficiency anaemia

## Abstract

Introduction

In spite of the implementation of the National nutritional anemia prophylaxis program in India, the prevalence of anemia during pregnancy is 50 in 100 women. One main cause for the high frequency of anemia among antenatal women in India is a lack of iron consumption. Few studies are available in the literature where efforts have been made to improve compliance with iron therapy. In the same line of thinking, it was decided in the present study to use mobile phone calls as a reminder for iron tablets.

Method

The present study is a randomized controlled open-label trial. Antenatal women with gestational age between 13-28 weeks of pregnancy diagnosed with iron deficiency anemia (Hb - 8 to 11 gm/dl) having mobile phones were included in the study. Recruited women were randomized into two groups of study groups receiving standard therapy with the addition of fortnightly mobile phone call reminders and a control group receiving standard therapy alone.

Results

Compliance with iron supplementation was observed higher in the study group as compared to the control group (range of 48% to 93%). There was a mean hemoglobin rise in both the groups at the time of delivery compared with the hemoglobin at the time of recruitment (Study group- 9.74 to 10.69, Control group- 9.48 to 10.06). There was a statistically higher (0.00001) increase in the mean hemoglobin concentration in the study group (0.96 gm/dl) as compared to the control group (0.59 gm/dl). The reasons for poor compliance were boredom with taking daily oral iron therapy (66.66%), constipation (3.7%), forgetfulness (14.81%), and heartburn (14.81%). There was no statistical difference in the reason for poor compliance with iron supplementation in both groups.

Conclusion

The present study concludes that mobile phone call reminders along with standard therapy with iron supplementation improve compliance with iron supplementation and lead to a greater rise in hemoglobin in antenatal women with iron deficiency anemia.

## Introduction

Anemia is a grave health-related problem in the world. The reported prevalence of anemia in antenatal women is more than 50% in India. Anemia adversely affects the quality of life by affecting physical and mental health. The National Iron+ Initiative guidelines were developed for preventing and controlling anemia in 2013. This initiative has adopted the lifecycle approach to cover all age groups. In India, anemia still causes 20% of death, postulated even after these efforts [1]. Despite the implementation of the NNAPP (National Nutritional Anemia Prophylaxis Program) in 1970, 50% of women in the reproductive age group still have anemia [1]. The Iron and Folic Acid Supplementation and Deworming program, National Iron+ Initiative, Anemia Mukta Bharat (intensified iron+ Initiative), and UNICEF-supported anemia-free future program were other initiatives taken to reduce the prevalence of anemia [2-5].

Anemia puts women of reproductive age at greater risk and interposes to a more significant number of maternal and perinatal mortality worldwide [6-7]. One leading cause of the high frequency of anemia among antenatal women in India is noncompliance with oral Iron supplementation [8-11]. Forgetfulness, constipation, boredom with taking oral tablets, and fear of side effects are the most common causes of poor compliance [12]. Few studies are available in the literature where efforts have been made to improve compliance to iron therapy using interventions like text messaging, directed observed intake, free medications, capsule form of iron, etc [13-19]. Regular oral iron treatment has been shown to reduce the prevalence of anemia in pregnant women [18]. Various studies have been carried out to calculate compliance with oral iron treatment [20]. Improving the patient’s and her family’s awareness about iron intake has resulted in better compliance with the treatment [21-25].

The mobile phone is recognized as a possible and feasible way of telemedicine, and it is readily available in nearly ninety-five percent of countries in the world that have mobile phone networks. SMS text messaging can also be used for delivering reminders for healthcare purposes like giving reminders for medication for asthma, diabetes, or other patients requiring chronic medication intake.

We planned this study to use mobile phone calls as a reminder for iron tablet consumption. Identifying the reason for poor compliance and encouraging women to adhere to iron therapy was considered an advantage over SMS texts. We started this study with the null hypothesis that there is no statistically significant difference in the effect of mobile phone call reminders with standard therapy compared to standard treatment alone on compliance with iron supplementation in antenatal women with iron deficiency anemia.

The study will also help to understand the causes of poor compliance in our population. It will help us to formulate future strategies to deal with anemia complicating pregnancy in our people.

## Materials and methods

This randomized controlled open-label trial was conducted at the Department of Obstetrics and Gynaecology at a tertiary care hospital. Antenatal women with gestational age between 13-28 weeks of pregnancy diagnosed with iron deficiency anemia (hemoglobin (Hb) 8 to 11 gm/dl) having mobile phones were included in the study. Women with hemoglobinopathies such as sickle cell disease, sickle cell trait, and thalassemia were excluded. Women with proven worm infestations were excluded from the study. Women not responding to mobile phone call reminders for four consecutive days were excluded from the study. Advia 2120 hematology system (Siemens Healthcare, Erlangen, Germany), a cyanide-free method, was used for hemoglobin estimation. After the institutional ethics committee approval, the study was conducted over two years, from 1st November 2019 to 31st October 2021.

Epi Info software version 7 (Centers for Disease Control and Prevention, Atlanta, USA) was used to calculate the sample size. The Collen formula was used to calculate the sample size. Based on the most recent Coverage Evaluation Survey results, we estimated compliance in the standard therapy group, i.e., the control group, to be 43%. We anticipate detecting an increase of 20% in the intervention arm (study group) at a 5% significance level, assuming a 15% attrition rate. Based on this, the total sample size required was 240.

Demographic data of each participant (age, parity) was recorded in case record form. Socioeconomic status was noted following the modified Kuppuswami classification [26]. General and obstetric examinations were done. Investigations were noted. Iron deficiency anemia was diagnosed based on complete blood count, peripheral smear report, and hemoglobin electrophoresis showing an AA pattern. Hemoglobin electrophoresis was done in all participating women to rule out hemoglobinopathies. Serum ferritin levels can also be used to diagnose iron deficiency anemia. Randomization was done by using computer-generated simple randomization with the help of GraphPad (GraphPad Software, San Diego, USA).

Recruited women were randomized into two groups: the study group, receiving standard therapy following the study guidelines with the addition of fortnightly mobile phone call reminders, and the control group, receiving standard therapy. A computer-generated simple random number table was used to allocate participants into two groups. According to the study protocol, IFA (iron folic acid) supplementation - 100 mg of elemental Iron and 500 mcg of folic acid - was given twice daily from recruitment until delivery.

Parenteral iron therapy is used in women with intolerance to oral Iron and a decrease in hemoglobin level compared to the level at recruitment. The dosage of iron sucrose was calculated in grams as per the formula - 2.3 x (11 - patient Hb) x pre-pregnancy weight + 1000 [27]. Calculated dosages were given in divided doses over one to two weeks. Oral iron was withheld for that period.

Hemoglobin was measured at least three times mandatorily for all women in the study as well as the control group. The first estimation was done at the time of recruitment, four weeks after starting the treatment, and then at the delivery time. 

All women were provided with iron (ferrous sulfate) tablets of 100 mg having 60 mg of elemental iron supplied free of cost by the government. Women were instructed to take iron tablets one hour before a principal meal with a twice-a-day dosage. Iron tablets were provided in a small plastic pouch, with 60 tablets being provided for one month. One month's medication was provided, and the exact dosage was repeated at monthly antenatal visits. Women with side effects such as nausea, vomiting, constipation, loose motions, and epigastric pain were asked to report earlier. Women were counseled about the importance of regular consumption of iron tablets and regular antenatal visits. Women were called every month if they were less than 32 weeks of gestation, fortnightly visits from 33 to 36 weeks, and weekly visits after 36 weeks of pregnancy. More frequent visits were advised in case of associated high-risk factors. Women in both groups were also counseled about the desirable hemoglobin level of ≥11 gm% during pregnancy. Dietary advice on iron- and protein-rich food and how to take the tablets was given. The possibility of ill effects of anemia on maternal and neonatal health was also explained to women in both groups. The same treatment protocol was followed in the study group. In addition to this standard therapy, mobile phone call reminders were given every 15 days.

Each time the women were asked whether they were taking two tablets of iron daily and how many tablets were remaining. The reasons for poor compliance were enquired about, and they were encouraged to take the tablets regularly.

In case of inability to contact by mobile phone, repeat phone calls were made on the following three consecutive days. In case of inability to contact the participant women for the next three days, the participants were withdrawn from the study. Women in both groups were followed till term. Compliance was ascertained by asking the number of tablets remaining at each follow-up or phone call. The compliance rate was calculated at the time of delivery of participating women with the help of the following formula [14].

Compliance = Number of tablets consumed/number of tablets prescribed × 100

Compliance was expressed in percentages. Compliance of ≥65% was considered good compliance. The reasons for poor compliance were asked during phone calls/at the time of subsequent antenatal visits.

Statistical analysis

The information was entered into Microsoft Excel (Microsoft Corporation, Redmond, USA) and analyzed with SPSS Version 20.0 (IBM Corp, Armonk, USA). Frequency and percentage were used to represent quantitative data. The Chi-square test was used to ascertain the relationship between two quantitative variables. A p-value of less than 0.05 was deemed statistically significant.

## Results

**Figure 1:**
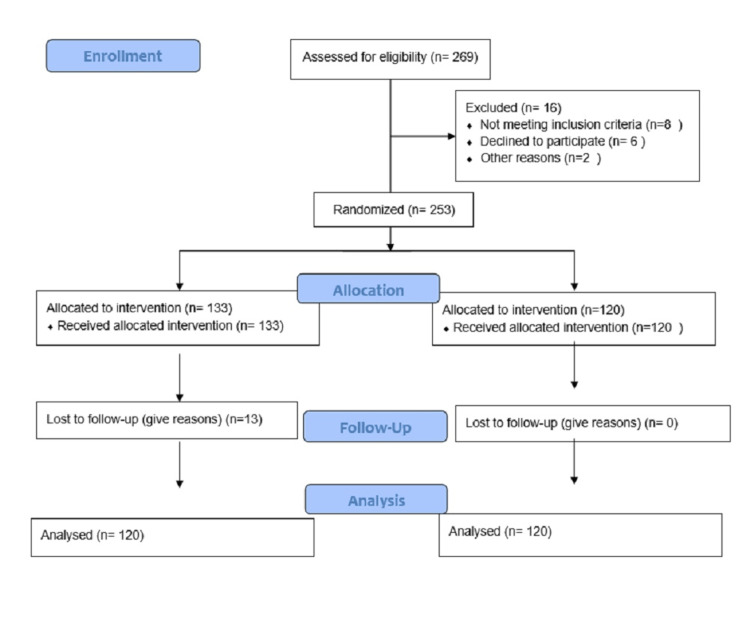
Recruitment and retention of the study population

The total number of women enrolled in the study was 253. Thirteen women in the study group could not be contacted by mobile phones on four consecutive days. As per the pre-decided methodology, these women were withdrawn from the study. The attrition rate in the present study was 5.13% (13 out of 253). For further analysis of the results, there were 120 females in the study group and 120 females in the control group (Figure 1).

Majority of participating women in both the groups were in the age group of 26-30 years (≤ 20- 5.41%, 21-25 - 36.25%, 26-30 - 40.41%, 31-35 - 14.41%, >35 - 3,33%). There was no relation between anemia with parity (p - 09489), age (p - 09489), and socioeconomic status (p - 0.4575) in both groups. The majority of women in both the groups belonged to lower middle socioeconomic status (upper middle - 27.91%, upper lower - 32.08%, lower middle - 37.5%, Lower - 2.5%). Additional parenteral iron therapy, wherever indicated, was given as per the pre-decided indication in nine women (Study group - 5, Control group - 4) (Table 01).

**Table 1:** Baseline characteristics of participants *Wani [26]

Baseline characteristics of participants			
	Study group	Control group	p-value
Mean Age	26.8 ±4.48	26.54±4.42	0.9489
Parity			
Nulliparous	60	63	0.9489
Multiparous	60	53	
Socioeconomic status (Modified Kuppuswamy classification)*			
Upper middle	29	38	0.455
Upper lower	38	39	
Lower middle	49	41	
Lower	4	2	
Routes of administration			
Oral	115	116	
Oral + Parenteral	5	4	

In the present study, the majority of participating women (81.66%) (ranging from 48% to 93%) had good compliance with iron supplementation. A greater proportion of women in the study group were compliant with iron supplementation (85.83%) than those in the control group (77.5%).

Better compliance for iron supplementation (≥65%) was observed in a greater number of participating women in the study group as compared to the control group (85.83% and 77.5% respectively). This difference was statistically significant (p - 0.0952) (Table 2).

**Table 2:** Comparison of compliance with iron supplementation between study and control groups

Route	study		Control		Total
	No of the study subjects	Percentage %	No of the study subjects	Percentage %	n (percentage)
Poor (<65%)	17	14.17	27	22.5	44 (18.33%)
Good (≥65%)	103	85.83	93	77.5	196 (81.67%)
Total	120	100.00	120	100.00	240
P-value	0.0952 Chi-square test (significant)				

There was a mean hemoglobin rise in both the groups at the time of delivery when compared with the hemoglobin at the time of recruitment (Study group - 9.74 to 10.69, Control group - 9.48 to 10.06). There was a statistically higher (0.0001) increase in the mean hemoglobin concentration in the study group (0.96 gm/dl) as compared to the control group (0.59 gm/dl) (Table 2). The reasons for poor compliance were boredom to take daily oral iron therapy (66.66%), constipation (3.7%), forgetfulness (14.81%), and heartburn (14.81%). The reasons for low iron supplementation compliance in the two groups were similar (Table 3).

**Table 3:** Comparison of increase in mean hemoglobin

Time of Hb level	study		Control		P-value (Unpaired t-test)
	Mean	SD	Mean	SD	
At the recruitment	9.74	0.68	9.48	0.68	
At delivery	10.69	0.89	10.06	0.7	
Change in Hb concentration	0.96	0.97	0.59	0.79	0.00001 (Significant)

## Discussion

Many studies were found in the literature that has studied iron compliance in pregnant women and the factors affecting it. The mean age of women was 26.8 years in the study group and 26.5 years in the control group (ranging from 26 to 30 years) which was similar to the previous studies [12-15].

Both groups had a comparable number of nulliparous and multiparous women. Khorshid reported similar findings [14]. Other studies by Ibrahim et al. have more nulliparous (54.4%) than multiparous (45.6%) [20]. In a study by Wiradnyani et al., there were more multiparous women than nulliparous women [23]. In the present study, most antenatal women fell in the upper lower and lower middle socioeconomic status (32.08% and 40.83%, respectively). Another study by Mithra et al. had antenatal women of lower socioeconomic status (72.1%) [12].

Various investigators have used different modes of intervention to improve compliance with iron supplementation, like direct observation of pill consumption [19], providing free tablets [17], SMS messaging [14], giving capsule formulation [13], and pill count [28]. Mobile phone call reminders were used to increase adherence to oral iron treatment. During the study period, nearly 25-30% of women in the study group discussed treatment modalities for minor pregnancy-related symptoms during reminder phone calls. It was noticed explicitly during the covid lockdown period when women were having difficulties reaching hospitals and also helped improve the doctor-patient relationship. Approximately 10% of the participating women called the researcher for health care advice even after they delivered.

Compliance with a medication regimen is commonly expressed as an adherence rate. Some studies used compliance [13-15], whereas some used adherence [19-20] to assess the patient’s behavior regarding the consumption of iron supplementation. There are no defined criteria to differentiate between good or poor compliance/adherence to iron supplementation during pregnancy. The cutoff point used by various studies to define good compliance/adherence is variable (65%/80%/90%). Few studies have used the mean compliance/adherence rate to compare the impact of the intervention.

In the present study, with the reminder phone calls as an intervention, there was a significant improvement in compliance in the study group when compared with the control group. Similar improvement with the various intervention was observed in all other studies [14-17] except in one study carried out by Srivastava et al. [13] in Odisha, India, where compliance was low in both groups. It might be because they have taken 90% of compliance as a reasonable compliance limit.

In the present study, a significant rise in Hb is observed at the time of delivery in the study group compared to the control group. Similar results have been found in other studies [15] except in a study conducted by Khorshid [14]. They conducted a randomized controlled trial on 116 pregnant women by giving them SMS reminders. They observed no significant change in the mean Hb of women after the intervention. Also, in a study carried out by Srivastava et al. [13] comparing tablet form and capsule form of iron preparation, there was no significant change in compliance. In the present study, most women's poor compliance was due to frustration with taking daily oral iron therapy. Apart from this, some other researchers have found vomiting, nausea, diarrhea, fear of side effects, and abdominal pain as other reasons for poor compliance with iron supplementation [14-15].

There were a few limitations of the study. We had a smaller number of participants in the study due to the COVID-19 lockdown in various parts of the country. The hemoglobin estimation may have been done more frequently to estimate the quantitative rise in the level with the duration of treatment. Around 80 percent of women have mobile phones in our region, so some of the women could not be included in the study. So mobile call reminders may not be useful for poor women who do not have mobile phones, and also for women living in remote areas with no mobile connectivity. The findings of the study can be generalized only to women living in urban areas having mobile phones and access to primary health care. 

## Conclusions

The present study concludes that mobile phone call reminders along with standard therapy with iron supplementation improve compliance with iron supplementation and, consequently, cause a greater rise in hemoglobin at the time of delivery in antenatal women with iron deficiency anemia as compared to standard therapy alone. Frustration to consume oral iron tablets daily, forgetfulness, heartburn, and constipation are the common reason for poor compliance in antenatal women with iron deficiency anemia, which needs to be addressed by educating women.
